# Editorial for the Special Issue “Infectious and Tropical Diseases: Symptoms, Diagnosis and Treatment”

**DOI:** 10.3390/medicina61030377

**Published:** 2025-02-22

**Authors:** Temi Lampejo

**Affiliations:** Department of Infection Sciences, King’s College Hospital, Denmark Hill, London SE5 9RS, UK; temi.lampejo@nhs.net

For centuries, humans have faced the devastating wrath of outbreaks, epidemics and pandemics caused by infectious diseases. Medical advancements, including vaccine developments, implementation of other preventative strategies and improved access to healthcare, have all helped to alleviate the global burden of infectious diseases. However, this has been accompanied by increased global connectivity and climate change, resulting in the emergence of infectious and tropical diseases and/or their vectors in regions of the world where they had previously been unseen [1]. An example of this is the recent multi-country Mpox (previously known as monkeypox) outbreak occurring across at least 16 countries in several parts of the world having previously been reported primarily in one continent [2]. The recent coronavirus disease 2019 (COVID-19) pandemic serves as a reminder that infectious diseases will continue to emerge and that the impact can be profound in both resource-rich and resource-limited settings. However, there still remains a disproportionate disease burden from infectious and tropical diseases in lower-income settings. Malaria, which predominantly affects lower-income regions of the world, was still responsible for an estimated 247 million new cases and 619,000 deaths in 2021 [3]. In the field of malaria, very positive recent advancements have been made in terms of the development of vaccines and monoclonal antibodies for prevention; however, there are many more infectious and tropical diseases that remain largely neglected in terms of preventative, therapeutic and diagnostic developments [4,5].

This Special Issue, comprising seven papers, covers a range of topics within the very broad and rapidly evolving field of infectious and tropical diseases, including unusual presentations of infections and the impact of infections on particularly ‘at-risk’ groups.

The first article in this Special Issue reports on the characteristics of COVID-19 in kidney transplant recipients and highlights the heterogeneous nature of COVID-19 disease in this patient population [6]. Rates of complications, intensive care unit (ICU) admission and death were shown to be significantly higher amongst kidney transplant recipients when compared to a control group without kidney transplantation. Importantly, a recent longitudinal study of solid organ transplant (SOT) recipients reporting on the evolution of COVID-19 outcomes across six waves of the pandemic found that COVID-19 outcomes significantly improved over time (although lung transplant recipients continue to have poorer outcomes relative to other SOT recipients) [7]. Despite these improvements, SOT recipients continue to remain at higher risk of severe disease compared to the general population.

The second article in this Special Issue describes a rare presentation of secondary syphilis in a patient with newly diagnosed type 2 diabetes mellitus and summarises the diagnostic and therapeutic approaches to syphilis [8]. Syphilis has affected humans for centuries, with the emergence of endemic syphilis being dated as far back as around 7000 BC [9]. Syphilis was associated with significant stigmatisation, morbidity and mortality. Penicillin was discovered in 1928 by Sir Alexander Fleming, and in 1943, it became the primary treatment for syphilis [9]. In more recent times, preventative, diagnostic and treatment programs globally have controlled its spread. There has, however, been a resurgence of syphilis in recent years in several parts of the world, highlighting the continued need for efforts to prevent ongoing transmission of this infection [10].

The third article in the Special Issue presents an unusual case of neurocysticercosis in a patient with longstanding HIV-1 infection, highlighting the diagnostic and therapeutic challenges of this neglected tropical disease [11]. Some diagnostic advancements have, however, been made with the development of highly sensitive and specific *Taenia solium* polymerase chain reaction (PCR) assays and novel portable fluorescent sensors for *T. solium* antibody detection [12]. In terms of treatment progress, evidence is emerging for the use of tumour necrosis factor alpha (TNF-α) inhibitors to reduce inflammation and its associated symptoms in patients receiving antiparasitic drugs [12]. A human vaccine for the prevention of neurocysticercosis is yet to be developed.

The fourth article in this Special Issue provides preliminary data demonstrating an increase in frailty in older adults following dengue infection, which can be severe and may persist, thus reinforcing the importance of access to rehabilitation services for these patients [13]. The epidemiology of dengue in the elderly population is recognised to be changing, with atypical clinical features being observed in this population, posing several management challenges [14]. This area is relatively understudied, and further work is needed, including additional data on the efficacy, safety, and most effective administration strategies for dengue vaccines in this vulnerable population.

The fifth article in this Special Issue, a prospective case–control study, investigated vitamin D levels and deficiency in individuals with tuberculosis (TB) versus healthy controls, demonstrating that vitamin D deficiency was more prevalent in patients with TB [15]. In their study, vitamin D levels showed no significant correlation with sputum acid-fast bacillary load in the pulmonary TB group. Although vitamin D deficiency has already been extensively studied in TB, with low serum vitamin D levels being associated with an increased TB disease risk (particularly amongst people living with HIV who have severe vitamin D deficiency), the vast majority of studies have focused on pulmonary TB [16]. In the few studies on extra-pulmonary TB, data are conflicting [16]. This study found lower vitamin D levels in the extra-pulmonary TB group compared to the pulmonary TB group, but this did not reach statistical significance [15].

Continuing with the tubercular theme, the sixth article in this Special Issue describes a very unusual presentation of TB: lingual TB in association with spinal TB (Pott’s disease) in the absence of symptomatic or radiographic features of pulmonary TB [17]. Oral TB is very rare, accounting for less than 1% of cases of extra-pulmonary TB. Their case serves as a reminder that TB can effectively present anywhere in the body and should be considered, particularly if there are epidemiological risk factors for TB. Their patient had a history of receiving the Bacillus–Guerin (BCG) vaccine and no known TB contacts, which also reinforces the fact that a diagnosis of TB should not be dismissed because of these factors. The immunosuppressive drugs that the patient was receiving likely contributed to the disseminated form of TB disease that developed. Despite being isolated over a century ago and the subsequent development of a vaccine coupled with significant advancements in diagnostics and anti-tuberculous therapy, TB still has a significant global health burden and continues to challenge us diagnostically and therapeutically [18].

The final article in this Special Issue discusses the potential role of haemoadsorption in the management of a severe pro-inflammatory illness secondary to a bite from the Loxosceles spider, an important public health issue around the world, particularly in Latin America [19]. The authors report a severe, life-threatening case of viscerocutaenous loxoscelism (VCL) with multi-organ dysfunction necessitating intensive care unit (ICU) admission. The patient survived following supportive management (including haemodialysis for severe renal impairment) along with corticosteroids, antimicrobials and haemoadsorption therapy. The authors highlight that there is no prior evidence for haemoadsorption therapy specifically in the management of loxoscelism but that it should be considered, and further studies are warranted. Extracorporeal haemoadsorption, for the removal of endotoxin and/or inflammatory cytokines, is increasingly being used in the ICU setting for the management of severe inflammatory states [20]. There are some data showing a reduction in blood cytokine levels, but evidence demonstrating a survival benefit in severe inflammatory disorders is lacking, and further study is required [20].

In summary, this Special Issue highlights the continued diagnostic and therapeutic challenges of managing infectious and tropical diseases and serves as a reminder of their ongoing high disease burden, particularly amongst at-risk and more vulnerable populations. It also demonstrates the significant advancements that have been and continue to be made in the field of infectious and tropical diseases.
